# Genetic Pattern and Demographic History of Orange‐Spotted Grouper (*Epinephelus coioides*) in the South China Sea by the Influence of Pleistocene Climatic Oscillations

**DOI:** 10.1002/ece3.70967

**Published:** 2025-02-12

**Authors:** Yongkun Chen, Zihao Luo, Zhichao Zhang, Zhisen Luo, Manting Ren, Xiongbo He, Hung‐Du Lin, Yunrong Yan

**Affiliations:** ^1^ College of Fisheries Guangdong Ocean University Zhanjiang China; ^2^ Guangdong Provincial Engineering and Technology Research Center of Far Sea Fisheries Management and Fishing of South China Sea Guangdong Ocean University Zhanjiang China; ^3^ The Affiliated School of National Tainan First Senior High School Tainan Taiwan

**Keywords:** demographic history, DIY‐ABC, *Epinephelus coioides*, genetic diversity

## Abstract

Orange‐spotted groupers ( *Epinephelus coioides* ) are commercially important fish species that inhabit coral reef areas and are distributed across tropical coastal regions throughout the Indo‐West Pacific Oceans. This study aims to assess the phylogeographic structure of *E. coioides* by analyzing 180 individuals collected from six locations along the coast of mainland China and Hainan Island, using the mitochondrial Cyt *b* gene and 17 microsatellite DNA markers. The mitochondrial DNA analysis revealed high haplotype diversity (0.882), while the microsatellite DNA data showed an average of 8.677 alleles among the six populations, indicating that all populations exhibit a high level of genetic diversity. The presence of two distinct evolutionary lineages of *E. coioides* , along with the lack of significant genealogical structures corresponding to sampling localities, suggests that isolation in marginal seas during glaciation with lower sea levels shaped their phylogeographic distribution patterns. The results from STRUCTURE, PCoA, and pairwise *F*_ST_ revealed significant genetic differentiation in the Lingshui region (LS population) compared to other populations, suggesting that the Lingshui region, adjacent to deep‐sea areas, remained isolated during glacial periods as it was not connected to the continental shelf of mainland China. Analysis of demographic history using ABC revealed that *E. coioides* experienced historical lineage diversification and admixture due to secondary contact.

## Graphical abstract

This study aims to assess the phylogeographic structure of *E. coioides* by analyzing 180 individuals collected from six locations along the coast of mainland China and Hainan Island, using the mitochondrial Cyt b gene and 17 microsatellite DNA markers. The results from STRUCTURE, PCoA, and pairwise FST revealed significant genetic differentiation in the Lingshui region (LS population) compared to other populations, suggesting that the Lingshui region, adjacent to deep‐sea areas, remained isolated during glacial periods as it was not connected to the continental shelf of mainland China.

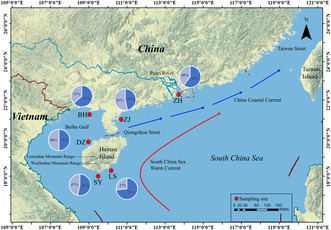

## 1 Introduction

Marine capture fisheries, coastal development, and pollution have contributed to population loss and a decline in genetic diversity in the ocean, primarily affecting within‐species variability (Dulvy, Sadovy, and Reynolds 2003). Conserving genetic diversity is a crucial objective of species conservation strategies and fisheries management, as it provides more opportunities for adaptation and resilience (Neff, Garner, and Pitcher 2011). In recent decades, concerns have risen about the overexploitation of marine resources due to overfishing, particularly for groupers, which are very popular in the South China Sea (Jiang et al. 2023; Kang et al. 2018). Replenishing or increasing the biomass of wild stocks through sea ranching, stock restoration, and stock enhancement is one of the important methods for conserving marine fish genetic resources (Grant et al. 2017). Removing a small number of individuals from the wild population for captive breeding may lead to a reduction in the effective population size of the supplemented wild stocks if these captive fish are offspring of only a few breeders (Araki, Cooper, and Blouin 2009). This artificial bottleneck can lead to the loss of genetic diversity in the combined captive‐wild system by increasing inbreeding and random genetic drift, as the enhancement of a portion of the wild gene pool can reduce the effective population size (*N*_e_), resulting in increased genetic drift and more rapid loss of genetic diversity, thus limiting evolutionary potential—a phenomenon known as the Ryman–Laikre effect (Waples et al. 2016). The exploitation of fisheries and aquaculture practices is exposing marine fish populations to increasing genetic risks, making it crucial to integrate genetic information into fisheries and aquaculture management to ensure the long‐term persistence of species (Lorenzen, Beveridge, and Mangel 2012).

In general, due to the lack of geographic barriers, marine organisms can be passively dispersed by ocean currents over long distances, leading to genetically homogeneous and panmictic populations (Han et al. 2022). However, the population genetic patterns and phylogeographic structures of some marine species are influenced by historical factors such as Pleistocene climatic oscillations, glacial vicariance, ocean current systems, and the life‐history traits of the organisms (Gu et al. 2022). China's coastal regions are characterized by marginal seas that were profoundly affected by Pleistocene glacial cycles. During glacial maxima, sea levels declined by 120–140 m, resulting in the formation of land bridges and the exposure of shallow continental shelves. These changes fragmented marine ecosystems and disrupted gene flow among aquatic populations. The sea‐level drop led to the semi‐closure of the South China Sea and exposed large portions of the East China Sea and Beibu Gulf. During the Pleistocene glacial–interglacial cycles in the northwestern Pacific, the emergence of the Taiwan Strait and Qiongzhou Strait, respectively, isolated the East China Sea, South China Sea, and Beibu Gulf (Gu et al. 2021; Qiu et al. 2016; Yang et al. 2022). This historical event has left a mark on many fish species in this region. For example, some marine fish, such as the Chinese black sleeper ( *Bostrychus sinensis* , Qiu et al. 2016), cutlassfish ( *Lepturacanthus savala* , Gu et al. 2021), and cutlassfish ( *Trichiurus nanhaiensis* , Gu et al. 2022), exhibit two distinct lineages due to vicariance events caused by the Taiwan Strait. Additionally, Yang et al. (2022) found that the Qiongzhou Strait has an isolating effect on the yellow grouper ( *Epinephelus awoara* ). However, numerous previous studies have shown that some highly mobile marine fish exhibit low genetic structure in the northwestern Pacific, such as the Chinese beard eel ( *Cirrhimuraena chinensis* , Li et al. 2014), ponyfish (*Nuchequula mannusella*, Gao et al. 2019), and golden puffer fish ( *Lagocephalus spadiceus* , Xu et al. 2024). Understanding the role of population genetic structure in species adaptation can provide valuable insights for the effective management and long‐term protection of marine species germplasm resources. Furthermore, the glacial–interglacial cycles also triggered extensive climatic fluctuations that significantly influenced species distributions and profoundly impacted the population dynamics of marine organisms. During glacial periods, repeated range contractions were followed by expansions into climatically favorable regions, known as glacial refugia. These cyclical population contractions and expansions played a critical role in shaping population dynamics and genetic diversity. Numerous studies have highlighted the importance of Pleistocene glacial periods in the demographic history of marine species, including *Monodonta labio* (Chiu et al. 2023), *Trichiurus nanhaiensis* (Gu et al. 2022), *Macrobrachium japonicum* (Han et al. 2022), and *Epinephelus awoara* (Yang et al. 2022).

Groupers (family Epinephelidae) are valuable and important marine fishes, facing high market demand and intense fishing pressure (de Mitcheson et al. 2020). They are particularly vulnerable to fishing pressure due to their life history characteristics, including longevity, late sexual maturation, and aggregation spawning (de Mitcheson et al. 2020). Orange‐spotted groupers ( *Epinephelus coioides* ) belong to the subfamily Epinephelinae within the family Serranidae. They inhabit coral reef areas and are distributed from eastern Africa, south to Durban, and eastward to the western Pacific, ranging from the Ryukyu Islands to Australia. *Epinephelus coioides* is a key commercial species farmed in South China. *Epinephelus* fish species are particularly susceptible to the loss of genetic diversity due to their specific life history traits, such as protogynous hermaphroditism, and their slow growth rate (Martinez, Willoughby, and Christie 2018). As a major resource, its populations may be difficult to recover once overfishing leads to a decline in genetic diversity.

Previous studies on the genetic diversity of *E. coioides* have primarily focused on characterizing microsatellite DNA and population structure using mtDNA. For example, an analysis of six populations along the coasts of Thailand and Indonesia, based on four microsatellite loci, revealed low genetic diversity and significant differentiation among them (Antoro, Na‐Nakorn, and Koedprang 2006). Similarly, a study using microsatellite technology on populations from Guangdong, Hainan, Malaysia, and Indonesia found significant differences among the wild populations, while the cultured populations showed reduced genetic diversity and significant differentiation (Wang et al. 2011). Tavakoli‐Kolour et al. (2022) analyzed wild populations in the Persian Gulf and the Sea of Oman using the D‐Loop molecular marker and found that their genetic diversity was similar to that of other grouper species. However, these studies often relied on single molecular markers or examined a limited number of populations, making it difficult to achieve a comprehensive understanding of the populations in Mainland China and Hainan Island.

Mitochondrial DNA is haploid and maternally inherited, reflecting changes in population genetic structure more quickly due to its lower effective population size and rapid coalescence time (Moritz 1994). Microsatellites, on the other hand, are simple repetitive sequences that are codominant throughout the eukaryotic nuclear genome, offering high resolution at the population level (Vieira et al. 2016). Each of these molecular genetic markers has its advantages. Therefore, this study employs both markers to investigate the population genetics and phylogeography of *E. coioides* populations in Mainland China and Hainan Island. Additionally, our results offer a viable and responsible framework for scientific and conservation efforts to ensure the long‐term sustainability and resilience of *E. coioides* resources against fishing pressure.

## 2 Materials and Methods

### 2.1 Sample Collection, DNA Extraction, and Microsatellite Genotyping

**FIGURE 1:**
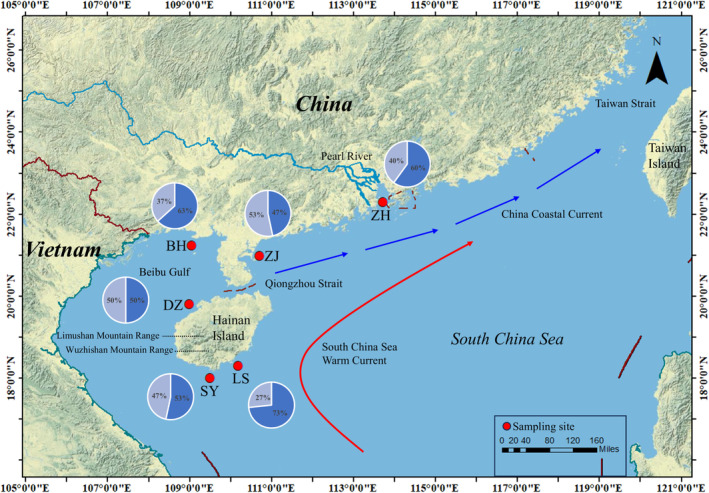
Map of the coast of Hainan Island and mainland China along the South China Sea illustrating the sampling locations of *Epinephelus coioides* . Each color in the pie charts represents the frequencies of the Lineage A (orange) and Lineage B (gray) haplotypes in each population. The sampling site/population code is described in Table 1.

A total of 180 *E. coioides* were collected from six locations in Hainan Island and mainland China along the South China Sea in 2023–2024 (Table 1, Figure 1). The samples were all caught by local fishermen through offshore fishing. All studies in animals were conducted according to ethical committee guidelines and approved by the Animal Research and Ethics Committee of the College of Fisheries, Guangdong Ocean University (China), and the study was carried out in compliance with the ARRIVE guidelines. After morphological identification, muscle tissues from all samples were stored in 95% alcohol for DNA extraction. Total DNA was extracted using a DNA extraction kit (Sangon Biotech, Shanghai, China).

**TABLE 1:** Sampling localities, code, latitude and longitude, sample size, genetic diversity for mitochondrial Cyt *b*, and microsatellite data of *Epinephelus coioides* .

Population	Code	Latitude and longitude	Sample size	mtDNA				Microsatellite				
				*N*_h_	*H*_d_	θπ (%)	θω (%)	*A*	*A*_r_	*H*_O_	*H*_E_	*F*_IS_
Ling Shui	LS	18.29° N 110.18° E	30	14	0.894	0.622	0.627	9.235	9.235	0.612	0.716	0.162
San Ya	SY	18.00° N 109.50° E	30	12	0.839	0.782	0.789	9.118	9.118	0.543	0.686	0.224
Dan Zhou	DZ	19.80° N 108.98° E	30	17	0.899	0.787	0.794	7.882	7.882	0.518	0.659	0.231
Zhan Jiang	ZJ	20.98° N 110.71° E	30	9	0.772	0.719	0.725	8.471	8.471	0.541	0.690	0.231
Bei Hai	BH	21.23° N 109.05° E	30	13	0.871	0.659	0.664	8.706	8.706	0.571	0.692	0.192
Zhu Hai	ZH	22.29° N 113.72° E	30	11	0.816	0.657	0.663	8.647	8.647	0.580	0.707	0.196
Clade A			104	21	0.776	0.134	0.554					
Clade B			76	24	0.758	0.130	0.501					
Mean			30	13	0.882	0.714	0.720	8.677	8.677	0.561	0.692	0.206

Abbreviations: *A*, average number of alleles; *A*_r_, allelic richness; *F*_IS_, mean level of inbreeding observed; *H*_d_, haplotype diversity; *H*_E_, mean expected heterozygosity; *H*_O_, mean observed heterozygosity; *N*_h_, number of haplotypes; θπ and θω, nucleotide diversity.

The upstream primer for the complete mitochondrial Cytochrome *b* (Cyt *b*) gene of *E. coioides* was designed as LM (5′‐GGCTTGAAAAACCATCGTTG‐3′), while the downstream primer is the universal primer H15915 (5′‐CTCCGATCTCCGGATTACAAGAC‐3′). PCRs were performed in 25 μL of the total volume containing 3 μL genomic DNA, 12.5 μL Taq mix (Sangon Biotech (Shanghai) Co.), 1 μL primer, and 7.5 μL ddH_2_O. PCR amplification was performed in a Biometra thermal cycler under the following conditions: 94°C for 5 min for initial denaturation, followed by 35 cycles of 94°C for 30 s, 58.5°C for 30 s, extension at 72°C for 2 min, and a final extension at 72°C for 10 min. All PCR products were purified and sequenced commercially by Sangon Biotech (Shanghai) Co. Ltd. Electrophoresis and genotyping were conducted by an ABI PRISM 3730 Genetic Analyzer automated DNA sequencer (Applied Biosystems Inc., Foster City, CA, USA) and GeneMapper 4.0 (Applied Biosystems). All nucleotide sequences were deposited in GenBank (accession numbers: PP993272–PP993451).

Thirty‐two microsatellite loci for this closely related species were previously developed and characterized (Wang et al. 2011). After screening these loci, we chose 17 polymorphic loci, namely Eco‐GSSR‐18, Eco‐GSSR‐17, Eco‐GSSR‐19, Eco‐GSSR‐10, Eco‐GSSR‐48, Eco‐GSSR‐45, Eco‐GSSR‐28, Mbo066, Pm‐12, Pm‐02, RH_CA_002, RH_CA_008, M2‐64, M2‐16, M4‐116, M3‐118, and M3‐33 for further analysis. The primers shown in Table S1 were subjected to PCR (MJ PTC‐200) amplification with a total volume of 10 μL, including 1.5 μL of template (100 ng/μL), 1 μL of 10× Buffer (Takara Bio Inc., Japan) (10 μM), 0.8 μL of dNTPs (10 mM), 0.5 μL of TaKaRa Taq DNA polymerase (Takara Bio Inc., Japan) (5 U/μL), 1 μL primer, and 5.2 μL of sterilized ddH_2_O. PCR program: 95°C denaturation 2 min; 40 cycles: 94°C denaturation 30 s, Ta (Table S1) 90 s, and 72°C extension 60 s; withdrawal cycle: 72°C extension 10 min.

### 2.2 Data Analysis

#### 2.2.1 Mitochondrial DNA Analysis

The Cyt *b* sequence alignments for all 180 samples were separately performed using Sequencher 5.4.5 software (GeneCode). The levels of intrapopulation genetic diversity were estimated based on indices of the number of haplotypes (*N*_h_), haplotype diversity (*h*), and nucleotide diversity (θπ and θω) using DnaSP v5.0 software (Librado and Rozas 2009). The phylogenetic relationship of *E. coioides* was analyzed using neighbor‐joining (NJ) in MEGA‐X (Kumar et al. 2018), Bayesian inference (BI) in MrBayes v3.1.2 (Huelsenbeck and Ronquist 2001), and maximum likelihood (ML) in IQtree v2.0 (Minh et al. 2020). The best‐fit partitioning strategy and nucleotide substitution model for BI and ML trees were selected by the HKY+F+G4 model using the Akaike Information Criterion (AIC_c_) in IQtree v2.0 (Minh et al. 2020). Haplotype networks for Cyt *b* were constructed using the median‐joining network method with Network 10.2.0.0 software (Bandelt, Forster, and Röhl 1999). The historical demographic expansions were examined using three different approaches. The neutrality tests (Tajima's *D* and Fu's *F*s) and mismatch distribution analyses were performed using DnaSP v5.0 software to evaluate the hypothesis of demographic expansion. Additionally, Bayesian skyline plots (BSPs) were generated using BEAST v2 software (Bouckaert et al. 2014) to analyze changes in the effective population size of *E. coioides* over time. The MCMC chains were each run three times for 100 million generations, with samples taken every 10,000 generations for each dataset. In the BSP's analysis, the effective sample size (ESS) values exceeded 200 for each parameter setting, and the 95% highest posterior density (HPD) intervals were reported. Bayesian skyline plots were analyzed and drawn using the Tracer v1.7.2 program (Rambaut et al. 2018). A previously estimated divergence rate of 1.05% per million years for the mtDNA Cyt *b* genes in Epinephelus fishes was used to study population expansion (Gu et al. 2022; Yang et al. 2022). The generation time of *E. coioides* is considered to be 2 years (Grandcourt et al. 2009).

Pairwise *F*_ST_ values between six population pairs and the analysis of molecular variance (AMOVA) were performed using Arlequin 3.5. Six populations were grouped with two scenarios based on geographical barriers: (1) Scenario I: two independent groups divided by the sea area (South China Sea group: LS, SY, ZJ, ZH; Beibu Gulf group: BH, DZ); (2) Scenario II: two independent groups divided by the Qiongzhou Strait (Mainland group: BH, ZJ, ZH; Hainan group: LS, SY, DZ). Additionally, an isolation‐by‐distance Mantel test was conducted using the ade4 package in R to examine the correlation between genetic differentiation and geographic distance (Dray, Dufour, and Chessel 2007). Geographic distances in kilometers between sampling sites were measured using the Google Earth database (http://earth.google.com) from GPS locations.

#### 2.2.2 Microsatellite DNA Analysis

All loci were tested for potential genotyping errors, null alleles, and allelic dropout using the Micro‐Checker ver 2.2.3 (Van Oosterhout et al. 2004). The number of alleles (*N*_a_), mean observed heterozygosity (*H*_O_), mean expected heterozygosity (*H*_E_), and deviations from Hardy–Weinberg equilibrium (HWE) for each population were calculated using Arlequin 3.5. Allelic richness (*A*_R_) and inbreeding coefficient (*F*_IS_) were calculated using FSTAT version 2.9.3. Population differentiation of *E. coioides* was analyzed by estimating *F*_ST_ and *R*_ST_ values with Arlequin and applying AMOVA procedures to the mitochondrial DNA data mentioned above.

Bayesian assignment tests using STRUCTURE v2.3.4 (Pritchard, Stephens, and Donnelly 2000) were applied to estimate the optimal number of homogeneous groups (*K*) and evaluate the degree of admixture among them based on microsatellite data (Pritchard, Stephens, and Donnelly 2000). For each value of *K*, ranging from 2 to 5, 10 independent runs were conducted, utilizing 100,000 iterations of the Markov chain Monte Carlo (MCMC) with a burn‐in period of 20,000 iterations. Structure Harvester Web 0.6.94 was used to determine the optimal number of genetic clusters within the data, utilizing the delta K and Evanno methods. GenAIEx version 6.503 was used to calculate allelic frequencies, and the R package “pheatmap” was utilized for data visualization and constructing allele frequency heatmaps. Moreover, to investigate the genetic relationship between populations, principal coordinate analysis (PCoA) was performed using the online software ChiPlot (https://www.chiplot.online) (Xie et al. 2023). A dendrogram was then created using the unweighted pair grouping method with arithmetic mean (UPGMA) based on Nei's genetic distance (1972), calculated using POPULATIONS ver. 1.2.28. The resulting dendrogram can be viewed in MEGA‐X (Kumar et al. 2018). To determine whether any of the populations had experienced a recent decline in effective population size, we compared excess heterozygosity with three models: the infinite allele model (IAM; Kimura and Crow 1964), the stepwise mutation model (SMM; Kimura and Ohta 1978), and the two‐phase model (TPM; Di Rienzo et al. 1994), which consists of 70% stepwise mutations and 30% infinite allele mutations. This analysis was conducted using Bottleneck 1.2.02 (Piry, Luikart, and Cornuet 1999). Given that 13 polymorphic loci were studied, the Wilcoxon signed‐rank test was appropriate for the data analysis (Piry, Luikart, and Cornuet 1999).

#### 2.2.3 Approximate Bayesian Computation (ABC) Analysis

**FIGURE 2:**
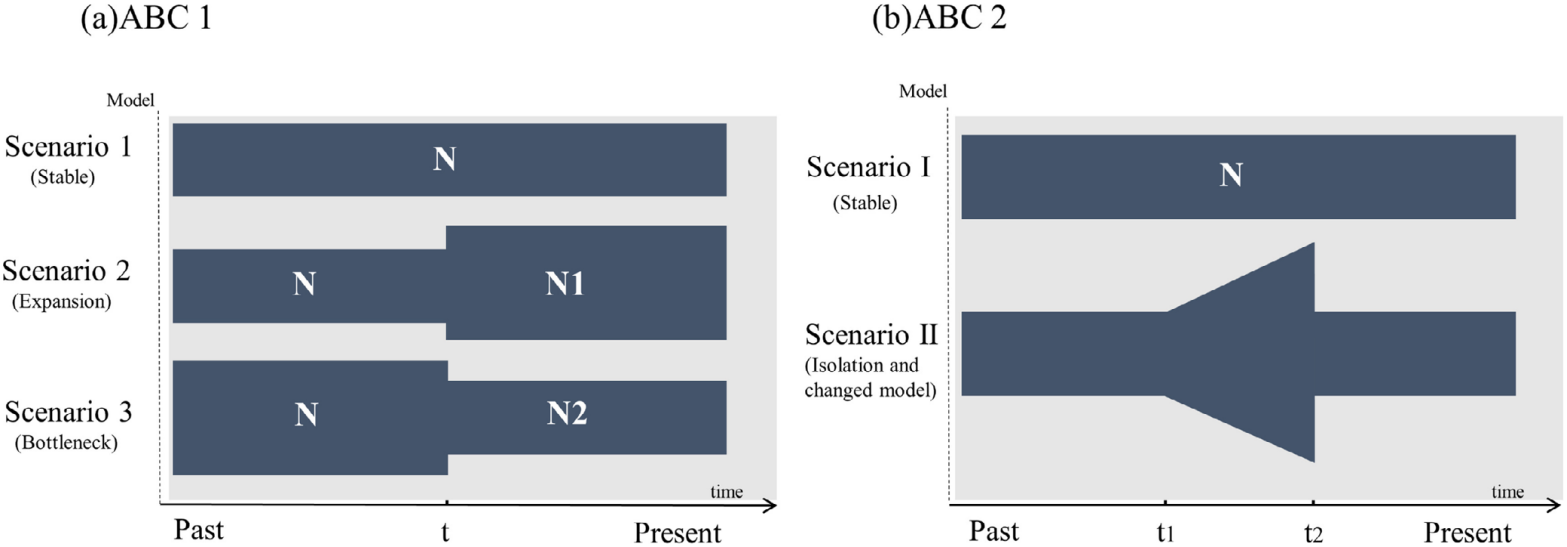
A schematic representation of five demographic scenarios for *Epinephelus coioides* tested using approximate Bayesian computation (ABC). Time and effective population size are not to scale. (a) Graphical representation of the three scenarios in the ABC 1 analysis; (b) Graphical representation of the two scenarios in the ABC 2 analysis.

To explore various historical and demographic evolutionary models, we employed approximate Bayesian computation (ABC) methods to address complex issues in *E. coioides* using DIYABC v.2.0.4 (Cornuet et al. 2014). We independently tested two levels of possible demographic scenarios for *E. coioides* using mtDNA Cyt *b* gene and microsatellite DNA markers. Our sequence dataset utilizes the HKY model as the best‐fit evolutionary model. The following scenarios were used: First, we used three different scenarios to test the changes in population sizes of *E. coioides* (ABC 1 analyses). Scenario 1 (CON model) is a stable size population, *N*_e_ (long‐term historical *N*_e_); scenario 2 (INC model) is a population that expanded recently, *N*_a_ (*N*_e_ during the expansion, *N*_e_ < *N*_a_); and scenario 3 (DEC model) consists of a population that is still experiencing a bottleneck, *N*_b_ (*N*_e_ during the bottleneck, *N*_e_ > *N*_b_). Secondly, we constructed two competing demographic scenarios for analysis using ABC 2. Scenario I represents a constant‐size population (long‐term historical *N*_e_), whereas Scenario II (Isolation and Change Model) involves population divergence and changes in effective population size during the periods t1 and t2. To obtain robust results, we performed a total of 3,000,000 simulations to generate the reference table for all demographic scenarios and calculated all summary statistics using the DIYABC software (Figure 2). The best‐supported scenario was determined by the highest posterior probability, calculated using a polychotomous weighted logistic regression on the 1% of simulated datasets closest to the observed dataset.

## 3 Results

### 3.1 Mitochondrial DNA

#### 3.1.1 Genetic Diversity

A total of 45 haplotypes were identified from 180 *E. coioides* individuals by sequencing 1141 bp of the complete mtDNA Cyt *b* gene, revealing a nucleotide composition of 14.5% guanine, 25.2% adenine, 28.3% thymine, and 32.0% cytosine, with a GC content of 46.5% (Figure 1, Table 1). Defining a total of 45 haplotypes, the study identified 15 shared and 30 private haplotypes. Among these, the most common haplotypes, Hap3 and Hap4, were shared by 35 and 42 specimens from six populations, respectively, representing frequencies of 19.4% and 23.3% of the individuals (Table S2). The average haplotype diversity of the mtDNA Cyt *b* gene was high (0.882), ranging from 0.772 (ZJ) to 0.899 (DZ), and the average nucleotide diversity (θπ) was low (0.007), ranging from 0.005 (DZ) to 0.006 (LS) (Table 1).

#### 3.1.2 Population Structure

The pairwise *F*_ST_ values ranged from 0.002 (between ZH and BH) to 0.039 (between LS and SY), with a mean value of 0.015 in the mtDNA Cyt *b* gene (Table 2). However, the pairwise *F*_ST_ values between the LS population and other populations were relatively high, ranging from 0.024 (LS and ZH) to 0.039 (LS and SY). This indicates significant differentiation among these groups. IBD analyses revealed no significant correlation between genetic distance and geographic distance (*r* = −0.141, *p* = 0.662). Hierarchical analyses of molecular variance (AMOVA) indicated that most genetic variation in the mtDNA Cyt *b* gene occurred among individuals within populations, with 99.30% in Scenario I and 98.83% in Scenario II (Table 3). Conversely, only −1.86% and −0.96% of the total variation were observed among the groups in Scenario I and Scenario II, respectively (Table 3).

**TABLE 2:** Matrix of pairwise *F*_ST_ among six populations based on microsatellite DNA (above diagonal) and mitochondrial Cyt *b* gene (below diagonal) in *Epinephelus coioides*.

	LS	SY	DZ	ZJ	BH	ZH
LS	—	0.039*	0.038*	0.034*	0.027*	0.024*
SY	0.036	—	0.004	0.002	0.016	0.021
DZ	0.046	−0.029	—	0.007	0.008	0.016
ZJ	0.094	−0.021	−0.022	—	0.010	0.015
BH	−0.001	0.015	0.015	0.057	—	0.002
ZH	0.013	0.007	0.004	0.042	−0.029	—

Abbreviations: BH, Beihai; DZ, Danzhou; LS, Lingshui; SY, Sanya; ZH, Zhuhai; ZJ, Zhanjiang.

* *p* < 0.015, modified FDR methods (Narum 2006).

**TABLE 3:** Analysis of molecular variance (AMOVA) for *Epinephelus coioides* populations based on mitochondrial Cyt *b* gene and microsatellite DNA.

Scheme	Category description	Variance components	Percentage variation	mtDNA		Microsatellite	
				*p*	Variance components	Percentage variation	*p*
I. Two groups (LS, SY, ZJ, ZH) (BH, DZ) [divided by the Qiongzhou Strait and Hainan Island]							
	Among groups	−0.075	−1.85	0.807	−0.018	−0.29	0.587
	Among populations within groups groups	0.103	2.56	0.134	0.097	1.60	**0.000**
	Among individuals within populations	4.019	99.30	0.167	1.234	20.30	**0.000**
	Within individuals				4.767	78.39	**0.000**
II. Two groups (BH, ZJ, ZH) (LS, SY, DZ) [divided by the Qiongzhou Strait]							
	Among groups	−0.039	−0.96	0.717	−0.006	−0.09	0.412
	Among populations Within groups	0.087	2.14	0.144	0.091	1.49	**0.000**
	Among individuals within populations	4.019	98.83	0.181	1.234	20.28	**0.000**
	Within individuals				4.767	78.32	**0.000**

*Note:* Significant scores (*p* < 0.05) are shown in boldface.

Abbreviations: BH, Beihai; DZ, Danzhou; LS, Lingshui; SY, Sanya; ZH, Zhuhai; ZJ, Zhanjiang.

#### 3.1.3 Phylogeny and Demographic History

**FIGURE 3:**
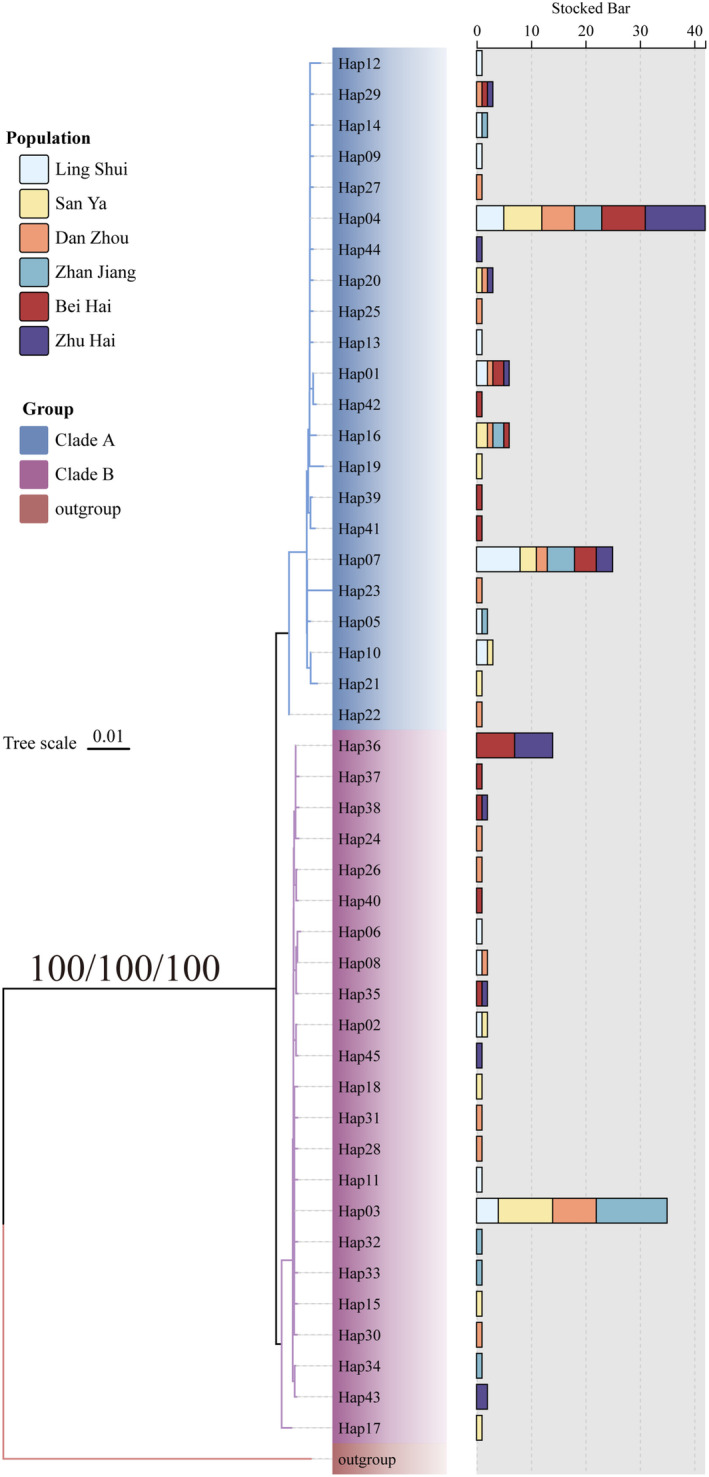
Phylogenetic trees reconstructed from mitochondrial sequences of the Cyt *b* gene in *Epinephelus coioides* . The values above the branches are bootstrap values for the NJ, ML, and BI analyses. Outgroups: *Epinephelus bruneus*.

**FIGURE 4:**
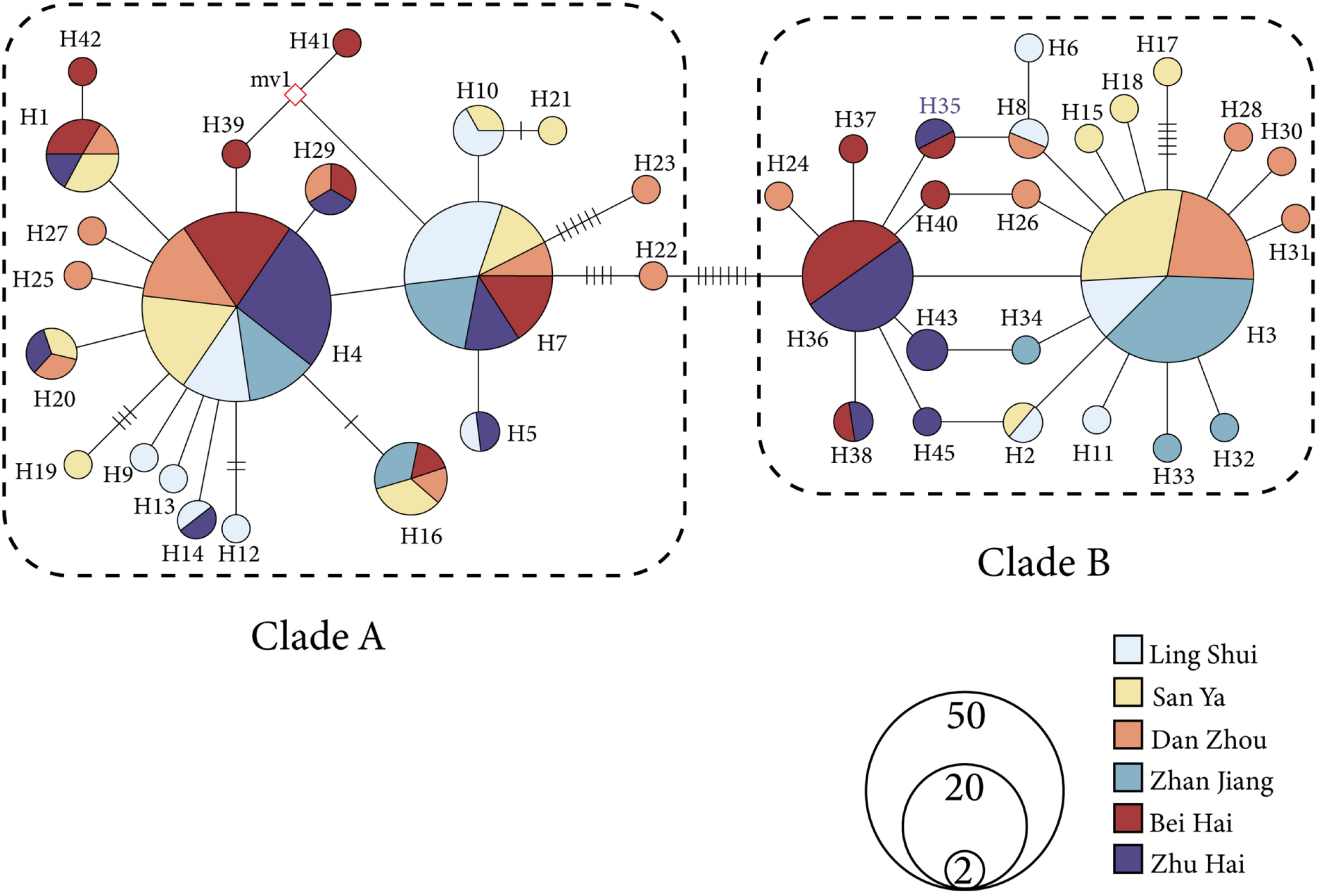
The phylogenetic relationships of *Epinephelus coioides* are represented in Median‐joining network based on mitochondrial Cyt *b* gene fragments. The haplotypes represent 180 individuals sampled from six geographic locations on the coast of Hainan Island and mainland China. Each circle indicates each haplotype, and the size of each circle is related to its haplotype distribution frequency. The red diamond represents a missing haplotype. Each branch connecting different circles represents a single nucleotide difference, and black crossbars represent an additional nucleotide difference. Dashed line rectangles indicate the lineage sorting results. Each color in haplotype network circles represents each geographic sample. Colors denote sample origins, and haplotype sizes correspond to distribution frequencies as indicated by the legends.

**FIGURE 5:**
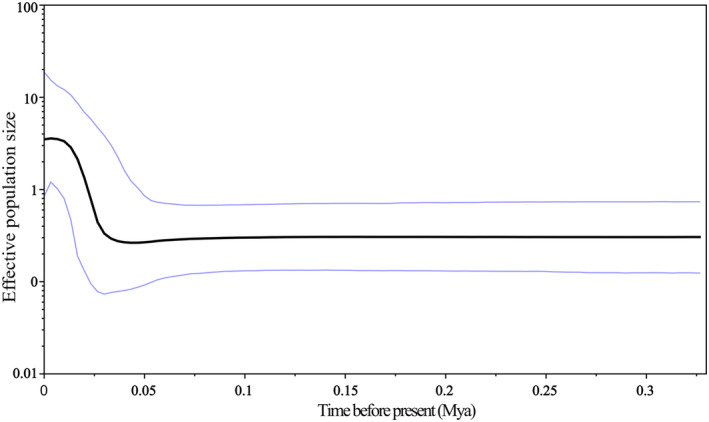
The Bayesian skyline plot illustrates the effective population sizes of *Epinephelus coioides* over time. The *y*‐axis represents the product of effective population size (*N*_e_) and generation length on a logarithmic scale, while the *x*‐axis shows the time before present, measured in millions of years ago.

Phylogenetic trees and haplotype networks based on the Cyt *b* gene indicate the formation of two major lineages, with genetic clusters not aligning with geographical sampling sites (Figures 3 and 4). Historical demographic events were analyzed using neutrality tests (Tajima's *D* and Fu's *F*s tests), mismatch distribution analysis, and Bayesian skyline plots to identify signatures. The nonsignificantly negative values of Tajima's *D* (−0.660, *p* > 0.05) indicate no signature of recent demographic expansion. However, the significantly negative values of Fu's *Fs* (−10.615, *p* < 0.05) suggest a deviation from neutrality, indicating a past population expansion for *E. coioides* 1997. Similarly, these findings were supported by the star‐like topology observed in the Cyt *b* gene tree. The Bayesian skyline plot indicated that *E. coioides* populations began a demographic expansion around 30,000 years ago, following a period of stable population history during the Pleistocene (Figure 5). The current (θω) and historical (θπ) genetic diversity values for lineages A and B were 0.501 and 0.130, and 0.554 and 0.134, respectively. When the current genetic diversity was larger than the historical genetic diversity in lineages A and B, the *E. coioides* populations revealed a pattern of decline. The bimodal distributions were revealed by the mismatch distribution analysis in *E. coioides* (Figure S1).

### 3.2 Microsatellite DNA

#### 3.2.1 Genetic Diversity

No indication of genotyping errors due to stutter bands, large allele dropout, or null alleles was revealed using Micro‐Checker ver 2.2.3 (van Oosterhout et al. 2004). A total of 232 alleles were detected for the 17 polymorphic markers, and the number of alleles per marker ranged from 6 to 32, with an average of 14, in 180 individuals, with 63 unique alleles in the total population. Table 1 presents the sample size, number of alleles (*N*_a_), allelic richness (*A*_R_) per population, expected heterozygosity (*H*_E_), observed heterozygosity (*H*_O_), inbreeding coefficient (*F*_IS_), and deviations from Hardy–Weinberg equilibrium (HWE) for each population. The average number of alleles and allelic richness for each population ranged from 8.200 (ZJ) to 10.600 (ST) (average = 9.050) and from 7.882 (DZ) to 9.235 (LS) (average = 8.677), respectively. The mean observed and expected heterozygosities were 0.561 and 0.692, ranging from 0.518 (DZ) to 0.612 (LS) and from 0.659 (DZ) to 0.716 (LS), respectively. The fixation index (*F*_IS_) in all populations was positive, indicating that the heterozygote deficiencies ranged from 0.162 (LS) to 0.231 (DZ and ZJ) (Table 1).

The characteristics of all 17 microsatellite loci in *E. coioides* are shown in Table S3. The average number of alleles (*N*_a_) and allelic richness (*A*_R_) per locus were 13.64 and 9.52, ranging from 6 (Pm‐02) to 32 (M2‐16) and from 4.08 (Pm‐02) to 21.07 (M2‐16), respectively. The observed heterozygosity (*H*_O_) and the expected heterozygosity (*H*_E_) were 0.739 and 0.723, ranging from 0.244 (Eco‐GSSR‐10) to 0.889 (Eco‐GSSR‐28) and ranging from 0.353 (Eco‐GSSR‐10) to 0.901 (RH_CA_002), respectively (Table S3). The average value of *F*_IS_ was 0.183, and most microsatellite DNA loci showed a significant heterozygote deficit and positive *F*_IS_ values, except Eco‐GSSR‐18 (−0.022), Eco‐GSSR‐48 (−0.002), and Pm‐02 (−0.084) (Table S3).

#### 3.2.2 Population Structure

The pairwise *F*_ST_ values ranged from 0.002 (between ZH and BH) to 0.039 (between LS and SY), with a mean value of 0.015, and the *R*_ST_ values, with an average of 0.207, ranged from −0.004 (between SY and ZH) to 0.622 (between LS and DZ) based on microsatellite DNA (Table 2). The pairwise *F*_ST_ values between the LS population and other populations were relatively high, ranging from 0.024 (between LS and ZH) to 0.039 (between LS and SY). Similarly, the pairwise *R*_ST_ values were also elevated, ranging from 0.596 (between LS and SY) to 0.622 (between LS and DZ) (Table S4). The results showed a nonsignificant correlation within IBD analyses between genetic differentiation and geographic distance using microsatellite DNA data (*r* = −0.300, *p* = 0.135). AMOVA showed that most of the genetic diversity was within individuals, i.e., two groups (Scenario I, 78.39%) and two groups (Scenario II, 78.32%) (Table 3). When the populations were divided into two groups (Scenario I) and two groups (Scenario II) according to the geographical barriers, only −0.29% and −0.09% of the total variation were found among the groups, respectively (Table 3).

#### 3.2.3 Phylogeny and Demographic History

**FIGURE 6:**
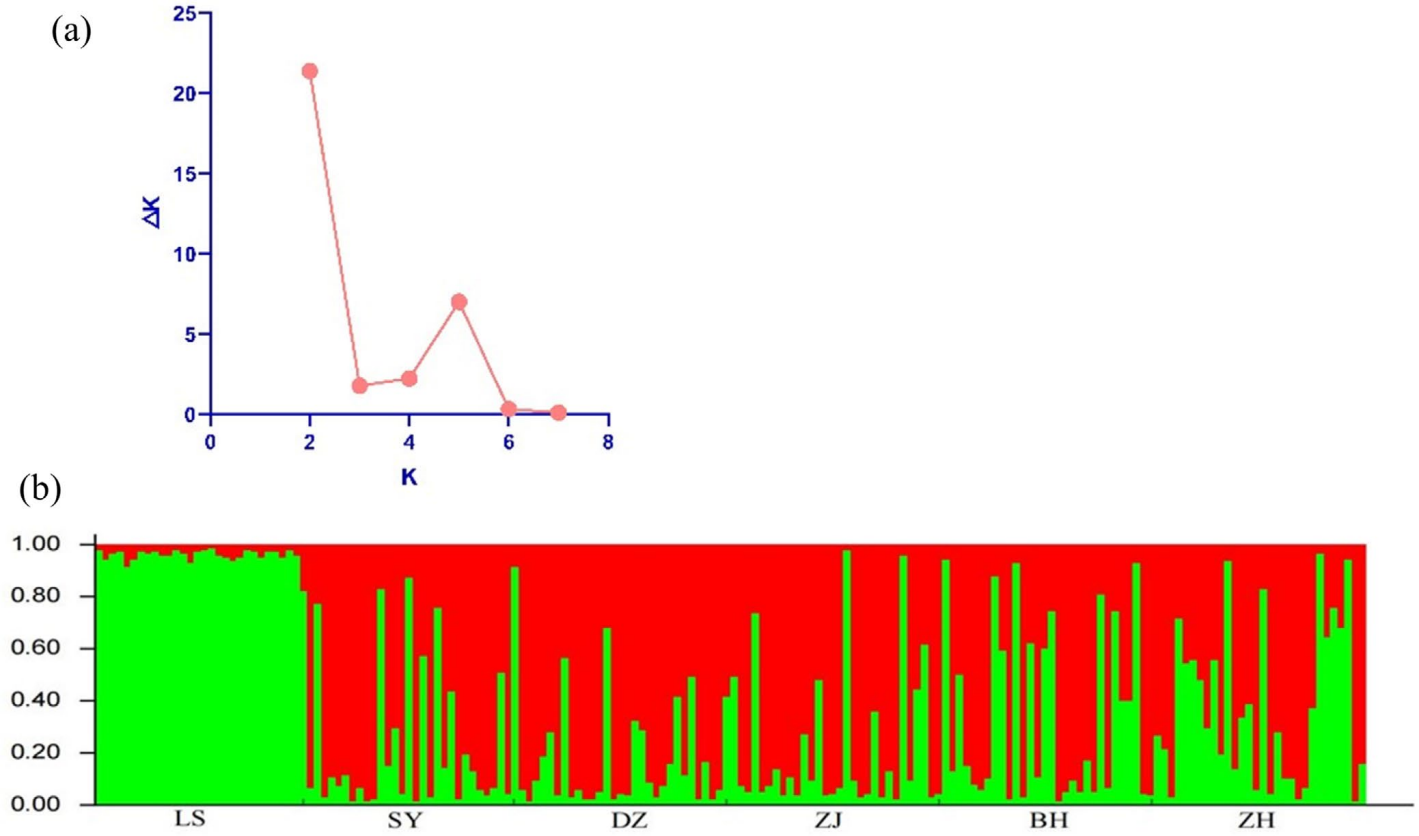
(a) Scatter plot of possible number of clusters (*K*; horizontal axis) against ad hoc statistic Δ*K* (vertical axis) based on the rate of change in the logarithm probability of the date between successive *K* values. (b) Clustering of individuals by structure at *K* = 2. Individuals are represented by vertical bars. Each vertical column represents one individual, and the separation of the column into two colors represents the estimated probability of belonging to one population or the other. Different colors in the same individual indicate the percentage of the genome shared with each cluster according to the admixture proportions. The *y*‐axis represents the probability of belonging to a certain cluster, while each population (code name given in Table 1) delimited by a black solid vertical line is reported on the *x*‐axis.

**FIGURE 7:**
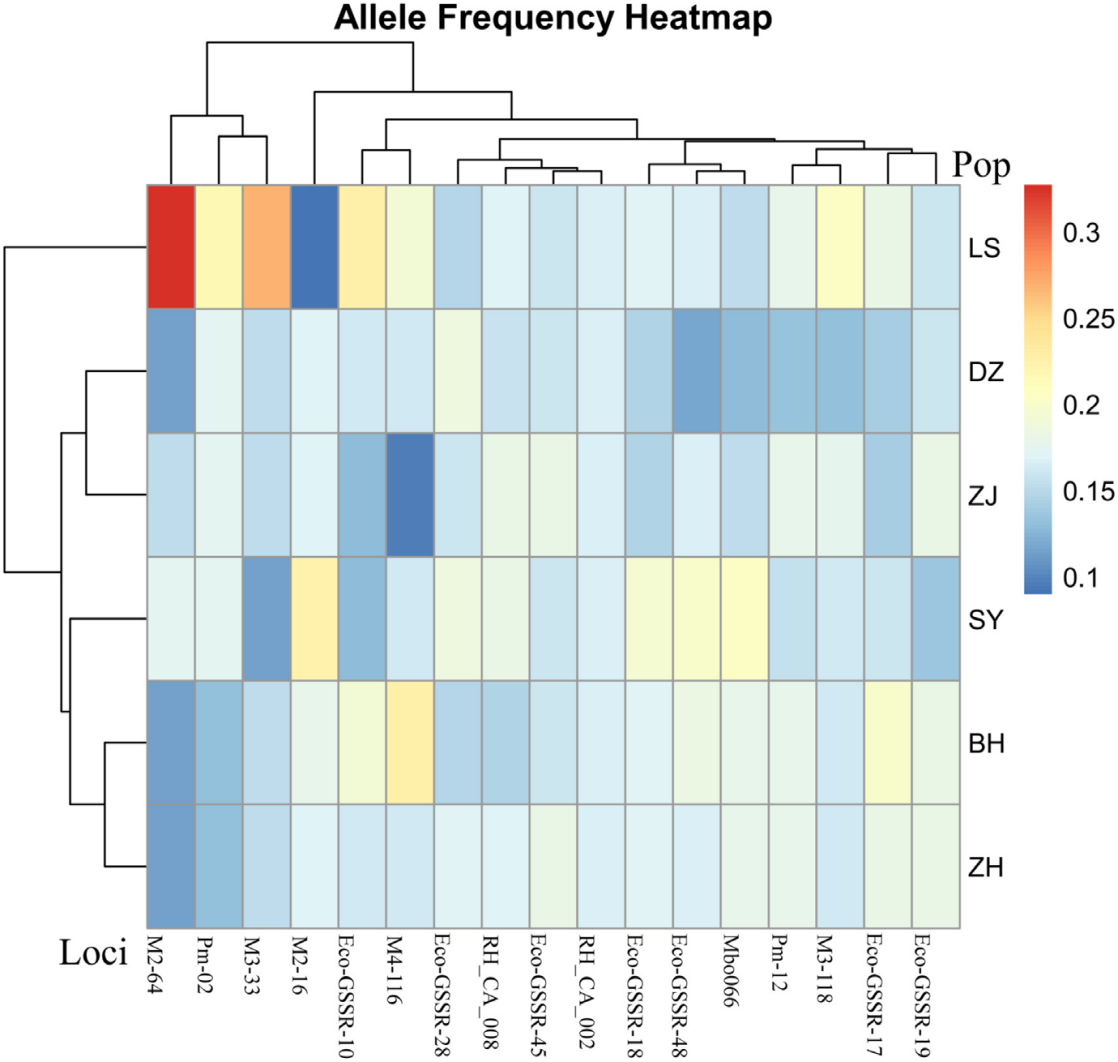
The heat map displays allele frequencies for 17 loci across all six populations. The color gradient from lighter blue to darker red indicates increasing allele frequencies, with red representing the highest allele frequency values. BH, Beihai; DZ, Danzhou; LS, Lingshui; SY, Sanya; ZH, Zhuhai; ZJ, Zhanjiang.

**FIGURE 8:**
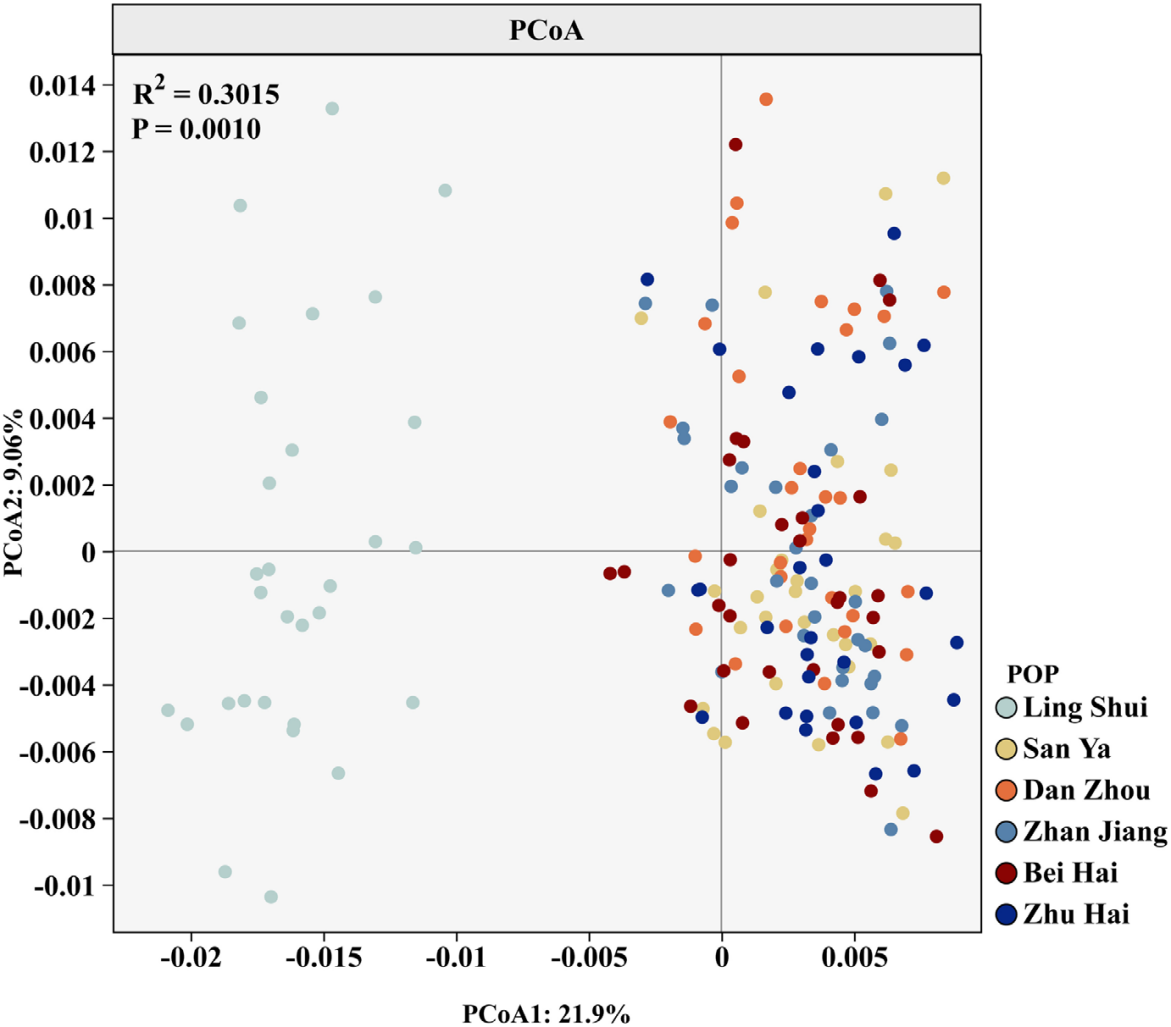
A scatter plot of the principal coordinates analysis (PCoA) showing relationships among six populations of *Epinephelus coioides* clusters based on 17 microsatellite loci. See Table 1 for the sampling sites/population codes.

We employed the Bayesian model‐based clustering algorithm in STRUCTURE 2.3.4 software to assign individuals to populations by estimating individual admixture proportions. This approach allowed us to explore different numbers of populations (*K*) in the population structure based on microsatellite data. The most likely number of clusters was determined using Structure Harvester, which revealed that all six populations separated into two distinct genetic clusters (*K* = 2, Ln *P*(*K*) = −10664.2; Stdev Ln *P*(*K*) = 11.253; Delta *K* = 21.397). The STRUCTURE analysis results supported *K* = 2, indicating that the populations of *E. coioides* could be divided into two distinct groups (Figure 6). The LS population belonged to one group, while the remaining populations formed the other group. Microsatellite DNA data were used to create a heatmap representing allele frequency for all 17 loci across six populations (Figure 7). The color intensity, as indicated by the color bar on the right, corresponds to allele frequency, with the firebrick indicating the highest values. The heatmap revealed that the LS population clustered at the top, while the remaining populations formed a separate group. Principal component analysis (PCoA) was conducted to verify the relationships among populations using genetic distances. The first two principal coordinates accounted for 21.90% and 9.06% of the variance, respectively, explaining 30.96% of the total variation. The PCoA results indicated that the populations of *E. coioides* could be divided into two groups, consistent with the STRUCTURE analysis. The LS population belonged to one group, while the remaining populations formed the other group (Figure 8). Phylogenetic tree construction following the UPGMA algorithm using the microsatellite DNA markers showed two groups, including the LS population, and the remaining populations formed the other group (Figure S2). The normal ‘L’‐shaped distribution of the mode‐shift test in all six populations suggested there was a stable population (Table S5). The Wilcoxon test showed no significant heterozygosity excess under both the TPM and SMM models, indicating that no genetic bottlenecks were detected in *E. coioides* due to mutation‐drift equilibrium. Therefore, the demographic history of *E. coioides* appears to be even more complex, and its populations have not experienced a recent genetic bottleneck (Table S5).

### 3.3 Approximate Bayesian Computation

The ABC analysis was conducted to explore the potential demographic history of *E. coioides* . In the ABC1 analyses, the “stable scenario” was strongly supported, with a posterior probability of 0.9189 (95% credible interval: 0.8179–1.0000), compared to the “expansion scenario” (posterior probability = 0.0206, 95% credible interval: 0.0000–0.0533) and the “bottleneck scenario” (posterior probability = 0.0605, 95% credible interval: 0.0000–0.1491). These findings indicate that the effective population size of *E. coioides* has remained constant over time. However, the results from DIY‐ABC contradicted those obtained from other methods. To address this, we applied competing demographic scenarios in the ABC2 analyses. Scenario II, representing the “Isolation and Changed Model,” had the highest posterior probability, nearing the upper limit at 0.9977 (95% credible interval: 0.9973–0.9981). This suggests that populations of *E. coioides* along the coast of mainland China experienced periods of isolation followed by subsequent mixing.

## 4 Discussion

### 4.1 Genetic Diversity

Monitoring and maintaining genetic diversity is crucial for the long‐term survival and fitness of species or populations (Vrijenhoek 1994). This is vital for biodiversity conservation and the sustainable use of biological resources. As an integral part of conservation biology, preserving genetic variation maximizes the ability of species to adapt to environmental changes (Sgrò, Lowe, and Hoffmann 2011). Many factors influence the genetic diversity, including environmental conditions, genetic drift within the population, the bottleneck effect, inbreeding, and life history traits (Amos and Harwood 1998). Epinephelinae holds significant economic value in marine fishing and aquaculture industries, but their populations in China are declining due to overfishing and habitat destruction (Dai et al. 2022). High haplotype diversity and low nucleotide diversity are commonly observed in Epinephelinae populations based on mitochondrial gene analysis (Yang et al. 2022). The result of *E. coioides* is consistent with previous studies in Epinephelinae, with overall high haplotype diversity (0.882) but low nucleotide diversity (0.007) (e.g., *E. coioides* , Wang et al. 2008; *E. coioides* , Waludin et al. 2018; *E. fasciatus*, Kuriiwa et al. 2014; *E. polyphekadion*, Ma et al. 2018; and *E. awoara* , Yang et al. 2022). High haplotype diversity, similar to that of most marine fishes along the coastlines of South China, suggests that marine organisms experience significantly large waves of offspring (e.g., *Nemipterus bathybius* , Yi et al. 2021 (*h* = 0.980); *E. awoara* , Yang et al. 2022 (*h* = 0.968); *Scatophagus argus* , Yan et al. 2020 (*h* = 0.870); *T. nanhaiensis* , Gu et al. 2022 (*h* = 0.989)). The nucleotide diversity of *E. coioides* in our study was higher than the 0.003 reported in a previous study conducted in the South China Sea (Wang et al. 2008), but lower than the 0.021 found in a study conducted in Malaysia (Waludin et al. 2018). In this study, the average expected heterozygosity (0.692) of *E. coioides* was relatively lower than the average expected heterozygosity of marine fish populations (*H*_E_ = 0.790). However, the values for the average number of alleles per population, observed heterozygosity, and expected heterozygosity were similar to those found in a previous study conducted in the South China Sea, yet relatively higher than the values reported in Thailand and Indonesia by Antoro, Na‐Nakorn, and Koedprang (2006). For *E. coioides* , the coastlines of South China are at the edge of its distribution range, while Malaysia is located at the central part of its distribution. Therefore, we believe that the high nucleotide diversity in the Malaysian population is because population size is considered to be an important factor in the maintenance of genetic variation, and small populations should show lower levels of genetic variability than large populations. The reason our study shows higher nucleotide diversity compared to previous studies is that we identified the LS population, which has a higher degree of differentiation, whereas previous studies did not. Consistent with previous studies, the positive inbreeding coefficient (*F*_IS_ = 0.145) of *E. coioides* indicates a deficiency in heterozygosity across all populations, likely due to inbreeding. We believe that the loss of genetic diversity may reflect past historical events in conjunction with current evolutionary forces, such as overfishing (Pinsky and Palumbi 2014), escaped individuals from aquaculture and artificial restocking (Yao et al. 2024), or habitat degradation (Airoldi, Balata, and Beck 2008). Genetic diversity loss from inbreeding depression is frequently associated with diminished adaptive capacity and fitness in populations. Therefore, maintaining high levels of genetic variability in natural populations is recommended for conservation.

### 4.2 Phylogeography and Population Structure of *Epinephelus coioides*

Marine organisms are generally expected to exhibit genetic homogeneity due to the high dispersal abilities of larvae or mature individuals, along with the apparent lack of significant barriers to gene flow between ocean basins in the coastlines of Hainan and mainland China (e.g., *C. chinensis* , Li et al. 2014; *Thamnaconus hypargyreus* , Wang et al. 2016; *L. spadiceus* , Xu et al. 2024). However, the historical processes linked to climatic oscillations during the Pleistocene ice ages are among the most crucial factors influencing the divergence of lineages in marine organisms (Qiu et al. 2016; Gu et al. 2021; Yang et al. 2022). A significant drop in sea level in the Taiwan Strait during the Pleistocene acted as a biogeographic barrier, potentially halting migration on either side and leading to the formation of two distinct lineages in marine fishes. As sea levels rose during the postglacial period, these lineages experienced secondary contact from different glacial refuges (Qiu et al. 2016; Gu et al. 2021; Yang et al. 2022). Our results indicate that haplotype networks and phylogenetic trees, based on the *Cyt b* gene, support the formation of two major lineages, while genetic clusters do not align with geographical groups. Many previous studies have shown the existence of two distinct genetic lineages, and the lack of phylogeographic structure in the South China Sea is consistent with our findings. This pattern has been observed in species such as *B. sinensis* (Qiu et al. 2016), *P. argentata* and *P. anea* (Lim, Habib, and Chen 2021), *T. nanhaiensis* (Gu et al. 2022), and *E. awoara* (Yang et al. 2022). The results of the AMOVA test, based on mitochondrial and microsatellite DNA data, indicated no significant differences at any hierarchical level. This suggests that the Qiongzhou Strait and Hainan Island do not act as geographic barriers. The results from STRUCTURE and PCoA showed two clusters when all populations were analyzed using microsatellite DNA data. This revealed genetic differences between the LS population and the other populations. The pairwise *F*_ST_ between the LS population and other populations was the highest among all comparisons, indicating the greatest genetic differentiation between the LS population and the others. This is a particularly interesting and unprecedented discovery among marine fish in the Lingshui region of Hainan Island (LS population). In this region, freshwater fish form a distinct geographic fauna on Hainan Island and the mainland of China, such as *Channa gachua* , Wang et al. 2021; *Opsariichthys hainanensis*, Wang, Wu, et al. 2022; Wang, Zhang, et al. 2022. Previous studies suggested that this phenomenon is related to marine topography. The Lingshui region is adjacent to deep‐sea regions, and during the glacial periods, this area was not connected to the continental shelf of mainland China, leading to prolonged isolation. We believe this factor also affects the *E. coioides* in this region. During the glacial periods, this area might have been an isolated bay, and the isolation effect resulted in greater genetic divergence of this population from other populations.

### 4.3 Demographic History and DIY‐ABC

Our results show that the nonsignificant negative values of Tajima's *D* (−0.660, *p* > 0.05) do not indicate any signs of recent demographic expansion. However, the neutrality indices from Fu's *F*s tests (−10.615, *p* < 0.05) suggest that *E. coioides* populations have experienced recent demographic expansion. Both the star‐like structure of the haplotype network and the Bayesian skyline plot consistently provide evidence of population demographic expansion. The Bayesian skyline plot estimates that the population demographic expansion of *E. coioides* occurred approximately 0.025 million years ago. Rising sea levels during interglacial periods inundated continental shelves and expanded marginal seas in the China Seas, significantly influencing the distribution and abundance of marine species (Avise 2000; Hewitt 2004; Liu et al. 2007). These environmental changes likely had profound genetic implications for population expansion (Gu et al. 2022; Wang, Wu, et al. 2022; Wang, Zhang, et al. 2022). Recent studies have identified similar patterns of population growth among various marine taxa in response to Pleistocene sea‐level fluctuations, consistent with previous findings on population expansion events in marine fishes from Hainan Island and Mainland China. These studies suggest that the population expansion occurred during the Last Glacial Maximum (LGM), as observed in species such as *T. hypargyreus* (Wang et al. 2016), *N. bathybius* (Yi et al. 2021), *E. awoara* (Yang et al. 2022), and *L. spadiceus* (Xu et al. 2024). However, a higher θω than θπ indicated population decline, the mismatch distribution seems to be bimodal, and some populations appeared to experience genetic bottlenecks based on the microsatellite markers in *E. coioides* . This demographic scenario of *E. coioides* reveals an even more complex history. Our best‐fitting ABC 2 model (Scenario II (the isolation and change model)) was identified with recent expansion preceded by a bottleneck. Compared with previous studies in the same sea area, the DIY‐ABC analysis also showed a similar expansion pattern. We believe that the significant drop in sea level in the Taiwan Strait during the Pleistocene acted as a biogeographic barrier, with subsequent secondary contact being the primary reason for this pattern. This is supported by studies on species such as *N. bathybius* (Yi et al. 2021), *T. nanhaiensis* (Gu et al. 2022), and *E. awoara* (Yang et al. 2022).

## 5 Conclusions

This study provides comprehensive insights into the genetic diversity, phylogeography, and population structure of *E. coioides* in the South China Sea. *Epinephelus coioides* populations exhibit high haplotype diversity (0.882) and low nucleotide diversity (0.007), consistent with other Epinephelinae species. Notably, the LS population in the Lingshui region of Hainan Island demonstrates significant genetic differentiation, likely due to historical isolation during the Pleistocene glacial periods. This geographic and genetic isolation underscores the importance of maintaining high genetic variability to ensure species adaptability and survival. The demographic history analysis indicates recent population expansion, preceded by a bottleneck event, aligning with patterns observed in other marine species post‐Last Glacial Maximum. The study highlights the necessity of conserving genetic diversity, particularly in light of overfishing and habitat destruction. Effective conservation strategies should focus on preserving natural population variability to enhance resilience against environmental changes. Overall, our findings emphasize the critical role of genetic diversity in the long‐term conservation of *E. coioides* and other marine species, advocating for targeted conservation efforts to mitigate the impacts of human activities and environmental changes.

## Author Contributions

**Yongkun Chen:** data curation (lead), formal analysis (lead), investigation (equal), methodology (equal), project administration (equal), software (lead), writing – original draft (lead), writing – review and editing (lead). **Zihao Luo:** methodology (equal), writing – review and editing (equal). **Zhichao Zhang:** writing – review and editing (lead). **Zhisen Luo:** writing – review and editing (equal). **Manting Ren:** writing – review and editing (equal). **Xiongbo He:** formal analysis (lead), investigation (equal). **Hung‐Du Lin:** methodology (equal), writing – review and editing (lead). **Yunrong Yan:** methodology (equal), writing – review and editing (lead).

## Conflicts of Interest

The authors declare no conflicts of interest.

## Supporting information


Appendix S1

## Data Availability

Data to support this study are available from the National Center for Biotechnology Information (https://www.ncbi.nlm.nih.gov, accessed on 9 July 2024). The registration numbers are PP993272–PP993451.
