# Supplementary material for: Genetic Pattern and Demographic History of Orange‐Spotted Grouper (Epinephelus coioides) in the South China Sea by the Influence of Pleistocene Climatic Oscillations
Source: Ecol Evol. 2025 Feb 12;15(2):e70967. doi: 10.1002/ece3.70967 (PMC11815338; doi:10.1002/ece3.70967)
Supplement: Supplementary file 1 — Appendix S1 [file ECE3-15-e70967-s001.docx]

Table S1. Characteristics of the 17 microsatellite DNA loci of *Epinephelus coioides.*

| Loci | Size of allels | TM(℃) | Repeat motif | GenBank No. |
| --- | --- | --- | --- | --- |
| Eco-GSSR-18 | 328-358 | 58 | (TAA)_7_ | KU255750 |
| Eco-GSSR-17 | 232-268 | 58 | (CCT)_10_(TCA)_9_ | KU255749 |
| Eco-GSSR-19 | 191-212 | 58 | (CTC)_7_ | KU255751 |
| Eco-GSSR-10 | 160-181 | 58 | (CCT)_10_ | KU255742 |
| Eco-GSSR-48 | 180-248 | 58 | (ATCA)_11_ | KU255780 |
| Eco-GSSR-45 | 230-270 | 58 | (CATA)_7_ | KU255777 |
| Eco-GSSR-28 | 317-381 | 58 | (ACTA)_11_ | KU255760 |
| Mbo066 | 91-111 | 58 | (CA)_7_ | AF325163 |
| Pm-12 | 187-211 | 58 | (TG)_9_ | EU117134 |
| Pm-02 | 160-172 | 58 | (CA)_7_ | EU117133 |
| RH_CA_002 | 112-148 | 58 | (AC)_27_ | EU117136 |
| RH_CA_008 | 198-236 | 58 | (CA)_18_ | EU117139 |
| M2-64 | 248-334 | 58 | (CA)_19_ | KT695778 |
| M2-16 | 246-328 | 58 | (TA)_10_ | KT695767 |
| M4-116 | 189-217 | 58 | (ATGG)_6_ | KU255772 |
| M3-118 | 123-171 | 58 | (TTA)_6_G(GTA)_15_ | KU255747 |
| M3-33 | 132-150 | 58 | (CCT)_10_ | KU255736 |

Table S2. The distribution information of the shared haplotypes (Hap01–Hap45).LS: Lingshui; SY: Sanya; DZ: Danzhou; ZJ: Zhanjiang; BH: Beihai; ZH: Zhuhai.

|  | LS | SY | DZ | ZJ | BH | ZH | total |
| --- | --- | --- | --- | --- | --- | --- | --- |
| P | 14 | 12 | 17 | 9 | 13 | 11 | 76 |
| Hap01 | 2 |  | 1 |  | 2 | 1 | 6 |
| Hap02 | 1 | 1 |  |  |  |  | 2 |
| Hap03 | 4 | 10 | 8 | 13 |  |  | 35 |
| Hap04 | 5 | 7 | 6 | 5 | 8 | 11 | 42 |
| Hap05 | 1 |  |  | 1 |  |  | 2 |
| Hap06 | 1 |  |  |  |  |  | 1 |
| Hap07 | 8 | 3 | 2 | 5 | 4 | 3 | 25 |
| Hap08 | 1 |  | 1 |  |  |  | 2 |
| Hap09 | 1 |  |  |  |  |  | 1 |
| Hap10 | 2 | 1 |  |  |  |  | 3 |
| Hap11 | 1 |  |  |  |  |  | 1 |
| Hap12 | 1 |  |  |  |  |  | 1 |
| Hap13 | 1 |  |  |  |  |  | 1 |
| Hap14 | 1 |  |  | 1 |  |  | 2 |
| Hap15 |  | 1 |  |  |  |  | 1 |
| Hap16 |  | 2 | 1 | 2 | 1 |  | 6 |
| Hap17 |  | 1 |  |  |  |  | 1 |
| Hap18 |  | 1 |  |  |  |  | 1 |
| Hap19 |  | 1 |  |  |  |  | 1 |
| Hap20 |  | 1 | 1 |  |  | 1 | 3 |
| Hap21 |  | 1 |  |  |  |  | 1 |
| Hap22 |  |  | 1 |  |  |  | 1 |
| Hap23 |  |  | 1 |  |  |  | 1 |
| Hap24 |  |  | 1 |  |  |  | 1 |
| Hap25 |  |  | 1 |  |  |  | 1 |
| Hap26 |  |  | 1 |  |  |  | 1 |
| Hap27 |  |  | 1 |  |  |  | 1 |
| Hap28 |  |  | 1 |  |  |  | 1 |
| Hap29 |  |  | 1 |  | 1 | 1 | 3 |
| Hap30 |  |  | 1 |  |  |  | 1 |
| Hap31 |  |  | 1 |  |  |  | 1 |
| Hap32 |  |  |  | 1 |  |  | 1 |
| Hap33 |  |  |  | 1 |  |  | 1 |
| Hap34 |  |  |  | 1 |  |  | 1 |
| Hap35 |  |  |  |  | 1 | 1 | 2 |
| Hap36 |  |  |  |  | 7 | 7 | 14 |
| Hap37 |  |  |  |  | 1 |  | 1 |
| Hap38 |  |  |  |  | 1 | 1 | 2 |
| Hap39 |  |  |  |  | 1 |  | 1 |
| Hap40 |  |  |  |  | 1 |  | 1 |
| Hap41 |  |  |  |  | 1 |  | 1 |
| Hap42 |  |  |  |  | 1 |  | 1 |
| Hap43 |  |  |  |  |  | 2 | 2 |
| Hap44 |  |  |  |  |  | 1 | 1 |
| Hap45 |  |  |  |  |  | 1 | 1 |

**Note:**P indicate the number of the private haplotypes in each population.

Table S3. Characteristics and genetic diversity indices for 17 microsatellite loci in *Epinephelus coioides*.LS: Lingshui; SY: Sanya; DZ: Danzhou; ZJ: Zhanjiang; BH: Beihai; ZH: Zhuhai.

| Locus | Na | PIC | Ar | Ho | He | Fis | Fst | Rst |
| --- | --- | --- | --- | --- | --- | --- | --- | --- |
| Eco-GSSR-18 | 10 | 0.688 | 6.918 | 0.739 | 0.723 | -0.022 | 0.013 | -0.006 |
| Eco-GSSR-17 | 11 | 0.688 | 8.37 | 0.422 | 0.706 | 0.402 | 0.026 | -0.002 |
| Eco-GSSR-19 | 8 | 0.724 | 7.449 | 0.544 | 0.741 | 0.265 | 0.017 | 0.004 |
| Eco-GSSR-10 | 8 | 0.356 | 5.938 | 0.244 | 0.353 | 0.308 | 0.040 | 0.013 |
| Eco-GSSR-48 | 15 | 0.738 | 9.77 | 0.756 | 0.754 | -0.002 | 0.012 | 0.007 |
| Eco-GSSR-45 | 10 | 0.523 | 7.01 | 0.528 | 0.534 | 0.011 | 0.018 | 0.001 |
| Eco-GSSR-28 | 17 | 0.902 | 13.402 | 0.889 | 0.894 | 0.006 | 0.016 | -0.005 |
| Mbo066 | 9 | 0.726 | 6.976 | 0.317 | 0.689 | 0.540 | 0.099 | 0.043 |
| Pm-12 | 12 | 0.539 | 7.289 | 0.511 | 0.560 | 0.087 | 0.009 | -0.009 |
| Pm-02 | 6 | 0.368 | 4.08 | 0.433 | 0.400 | -0.084 | 0.018 | 0.012 |
| RH_CA_002 | 17 | 0.904 | 13.589 | 0.800 | 0.901 | 0.112 | 0.011 | 0.001 |
| RH_CA_008 | 18 | 0.864 | 13.363 | 0.694 | 0.863 | 0.196 | 0.013 | -0.008 |
| M2-64 | 29 | 0.833 | 15.127 | 0.439 | 0.788 | 0.443 | 0.072 | 0.765 |
| M2-16 | 32 | 0.918 | 21.07 | 0.472 | 0.863 | 0.453 | 0.065 | 0.668 |
| M4-116 | 7 | 0.458 | 5.081 | 0.461 | 0.492 | 0.063 | 0.012 | -0.001 |
| M3-118 | 16 | 0.846 | 11.493 | 0.839 | 0.851 | 0.014 | 0.011 | 0.010 |
| M3-33 | 7 | 0.602 | 4.927 | 0.444 | 0.649 | 0.315 | 0.020 | 0.030 |
| mean | 13.647 | 0.687 | 9.521 | 0.739 | 0.723 | 0.183 | 0.028 | 0.090 |

Note: Na represents the observed number of alleles;PIC represents the polymorphic information content；Ho represents the observed heterozygosity; He represents the expected heterozygosity.

Table S4. Matrix of pairwise R_ST_ based on microsatellite DNA (above diagonal) and the corresponding P-value (below diagonal) in *Epinephelus coioides*.LS: Lingshui; SY: Sanya; DZ: Danzhou; ZJ: Zhanjiang; BH: Beihai; ZH: Zhuhai.

|  | LS | SY | DZ | ZJ | BH | ZH |
| --- | --- | --- | --- | --- | --- | --- |
| LS | - | 0.596* | 0.622* | 0.599* | 0.620* | 0.620* |
| SY | 0.000 | - | 0.005 | 0.002 | -0.003 | -0.004 |
| DZ | 0.000 | 0.261 | - | 0.007 | 0.022* | 0.005 |
| ZJ | 0.000 | 0.955 | 0.396 | - | 0.009 | 0.002 |
| BH | 0.000 | 0.649 | 0.045 | 0.144 | - | -0.001 |
| ZH | 0.000 | 0.757 | 0.288 | 0.414 | 0.649 | - |

Table S5. Bottleneck test of six geographic groups in Orange-spotted grouper.LS: Lingshui; SY: Sanya; DZ: Danzhou; ZJ: Zhanjiang; BH: Beihai; ZH: Zhuhai.

|  |  | LS | SY | DZ | ZJ | BH | ZH |
| --- | --- | --- | --- | --- | --- | --- | --- |
| IAM | p (one-tail for H excess) | 0.14208 | 0.21534 | 0.16447 | **0.03571** | 0.20188 | **0.03191** |
| TPM | p (one-tail for H excess) | 0.86834 | 0.95081 | 0.77071 | 0.57318 | 0.84698 | 0.84698 |
| SMM | p (one-tail for H excess) | 0.99808 | 0.99667 | 0.98992 | 0.99808 | 0.99768 | 0.99958 |
| Allele frequency distribution | | Normal L-shaped | | | | | |

The value of p <0.05 is in bold for Neutral test and Wilcoxon test.


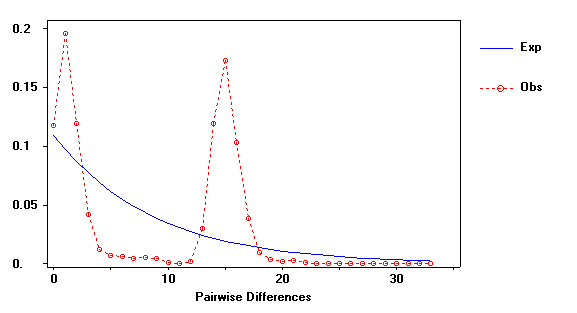


**Figure S1.** The bimodal distributions revealed by the mismatch distribution analysis in *Epinephelus coioides*.


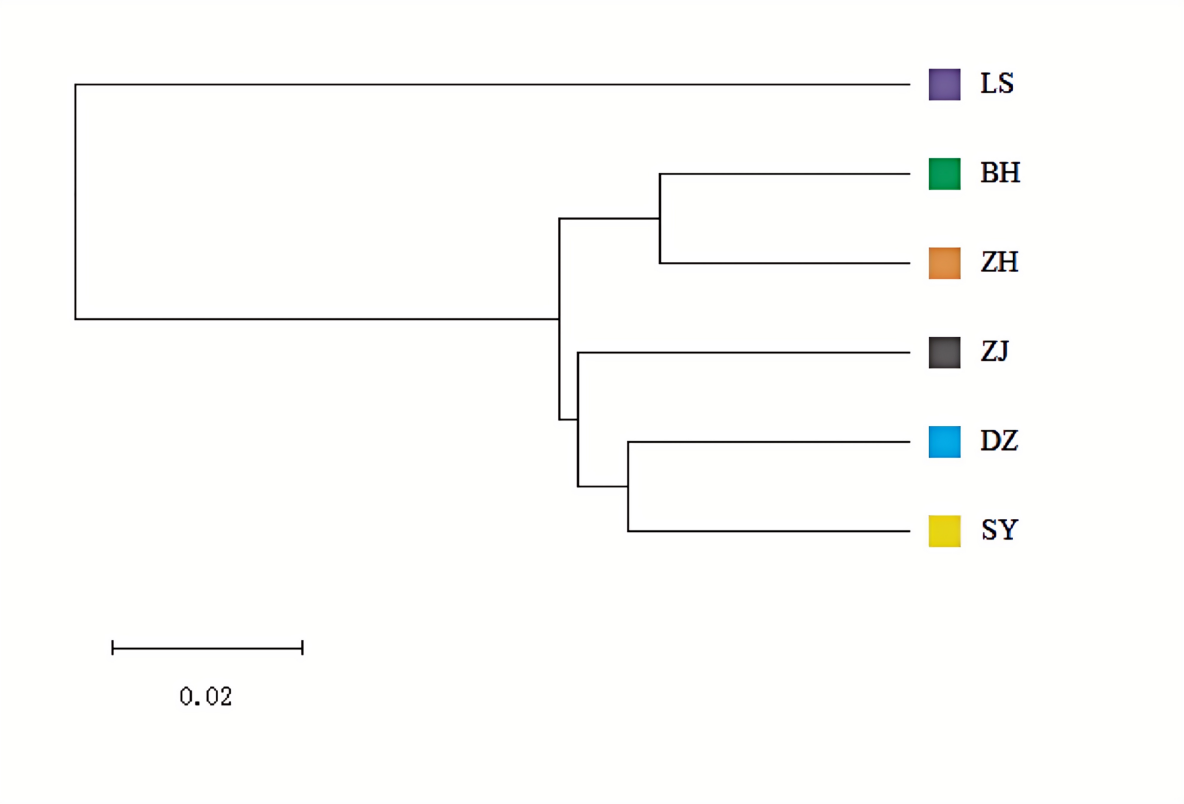


**Figure S2.** An unweighted pair group method with arithmetic means (UPGMA) tree based on Nei’s genetic distances of *Epinephelus coioides* populations.LS: Lingshui; SY: Sanya; DZ: Danzhou; ZJ: Zhanjiang; BH: Beihai; ZH: Zhuhai.
